# Total Ablation of the Inferior Maxilla, Executed with Success

**Published:** 1858-07

**Authors:** M. Maisonneuve

**Affiliations:** Surgeon to the Hôpital de la Pitié. Paris


VOL. III.] JULY, 1858. [No. XI.

### ORIGINAL COMMUNICATIONS.

### ARTICLE 1.

*Total Ablation of the Inferior Maxilla, executed with success.* By M. MAISONNEUVE, Surgeon to the Hôpital de la Pitié. Paris, 30th of June, 1857.

**Translated by T. A. Means, M. D., formerly of Georgia, now of Memphis, Tenn.**

"For several years past, the total ablation of the Inferior Maxilla was considered so dangerous an operation, that no French Surgeon dared to attempt it. Besides the extreme difficulties to be anticipated in the mere performance of the operation, in the neighborhood of both the internal and the external carotids, and the dangers to be apprehended from obstructed respiration, one was persuaded that after the mutilation of the tongue and its intimate attachments, anteriorly, it would necessarily be drawn backwards, towards the thorax, and thereby produce suffocation.

It was also believed—supposing the cure a probable one—that such a mutilation would leave the patient in a most lamentable condition, depriving him both of speech, and the power of mastication. Such was the general opinion when in the fall of 1853, I presented to the Académie, a young girl upon whom I had practiced a total ablation, and from which none of the accidents spoken of above, occurred. On the contrary, the most happy results were obtained.

In the summer of 1856, I had occasion to perform anew, the same operation upon a youug man, whom I afterwards presented to the Académie. In this, as in the preceding case, the operation was rapid and easy, and no accident intervened to prevent a speedy cure. The features were preserved in form and regularity—phonation was full and natural, and with the aid of a set of artificial teeth, he can chew the most solid food. He now occupies a position as counting master, in an iron manufactory in Spain. As an operation of such severity had thus been twice performed, it might be supposed that that circumstance alone would enable us to lessen the inconveniences and dangers attendant upon it. Neither the one last mentioned, however, nor the one now under consideration, have any striking features which demand special notice, and I shall add nothing of importance to what has been already furnished. The three cases taken together, however, so completely establish the practicability and safety of the operation, as to need no further confirmation, and may serve as an index from which results of the highest consequence may hereafter be obtained.

On the 23d of June, 1857, a young girl, aged 18 years, named Matilda S———n, came to consult me, in company with her mother, in relation to a voluminous tumor, situated upon the lower jaw. Its first development had scarcely been perceived, nor considered of much moment, yet within two months its growth became so rapid, that it caused her much alarm; but owing to the limited circumstances of the family, she could not consult a physician. At the end of twelve months she entered the hospital. The development of the tumor was so rapid, that it had already embraced the whole of the right side, involving, to some extent, the superior third of the ramus, and upon the left, had reached a point on a line with the three molars. It was the seat of lancinating pains which came on at intervals of three or four hours—the teeth were complete, save the first molar, (left,) which had been partially withdrawn: the soft parts were perfectly healthy.

After a thorough examination, I was convinced that the seat of disease was in the osseous tissue itself; that there would be consequently serious inconveniences if left to itself, or if a section only were made, there would, in all probability, be a return of the disease in a more aggravated form. Besides, if the small portion of the healthy bone were left, it would be of little use to the patient.

Considering all these inconveniences, I proposed to remedy the existing evil by a total disarticulation, to which the mother of the girl consented. The operation was performed 30th June, viz:

1st. After the usual anesthetic (chloroform,) was given, and *partial* anasthesia produced, (for I was fearful if *complete*, strangulation by blood would ensue;) I made a vertical incision upon the median line from the superior margin of the lip (inferior,) *to the bone* in front, and two inches down the margin of the genio-hyoideus muscle.

2d. By means of a chain saw, I divided the bone on a line with the symphysis, thus enabling me to establish with certitude, the nature of the disease.

3d. With the point of my bistoury, I separated rapidly the periosteum with the ends of my fingers, and when reaching the muscles, (masseter and pterygoideus) I tore them violently and in like manner the dental nerves and vessels.

5th. With the point of my curved scissors, I cut the still adherent cartilaginous insertion of the pterygoideus muscle, at its sphenoid origin.

6th, and lastly. By a brisk rotary movement, I disarticulated the bone, tearing all the articular ligaments of the internal pterygoideus muscle.

The first act of the operation having been completed, I then proceeded to disarticulate the left side, which was executed rapidly, and in like manner. What was most remarkable, after the operation I had not the least occasion to use the ligature. The pterygoid and masseteric branches of the facial artery, together with all the smaller arteries in that region, did not emit so much as *a drop of blood.* The inferior labial, and coronary arteries, which were necessarily divided by the first incision with the bistoury, ceased to bleed almost immediately—at least, some time before completing the operation, and entirely so, upon bringing the two halves in juxtaposition, by the twisted, or figure of 8, suture. The tongue, retained by its insertion to the periosteum, had not the least tendency to fall back into the thorax, and the movements of deglutition commenced ten hours afterwards. In fine, no accident intervened, and the cure was complete fifteen days afterwards.

Six weeks from the date of the operation, the features had recovered their form and regularity; and, save mastication, all the other functions of the mouth were performed as if the patient had never undergone the operation.

Remarks—by the translator.

Here are three cases of a total ablation of the inferior maxilla, followed by the most happy results, and totally exempt from accident and deformity, which heretofore have been so much dreaded by the surgeon. Convinced, therefore, of the value of the foregoing *facts*, by personal observation, I venture the following conclusions:

1st. That the total ablation of the inferior maxilla may be performed with safety, and unattended with inconveniences and dangers.

2d. That it does not hazard any important function.

3d. That it does not necessarily produce a sad deformity.

4th. That all the soft parts may be preserved "in situ," and by the new osseous deposit from the periosteum—as indicated by the Professor—secure to the patient *geneives* of sufficient firmness to answer all practical purposes.

5th. That it should merit—in this respect—a position in surgery as a regular operation.

I witnessed the operation reported, as well as watched the progress of the cure from the following morning until 22d of August, at which time the patient quitted the hospital.

The young girl in question is a brunette, of delicate frame, but of good constitution, with features regular and rather handsome. The Professor, when commencing the operation, stated his intention to disarticulate both extremities without making an incision, but he found, after having dissected back the periosteum on each side to the first molars—that the mouth was not possessed of sufficient elasticity to admit of further extension.

He resolved, therefore, to make one perpendicular incision instead of the crucial, or arched, as indicated in the books.—The bistoury was used only in the first incision, the remainder of the operation was performed with the ends of his fingers. All the smaller arteries, consequently, bled but little, and the hemorrhage observed was altogether venous—Anasthesia ceased within five minutes after the first stroke of the bistoury, and the patient submitted to the remainder of the operation without a murmur.

I should remark, that the operations mentioned were performed after the method *sons periostique*, the principles of which were first advanced and claimed by M. Flourens.

No other method would admit of an execution so rapid, and at the same time so secure; none similar, in which the hemorrhage can be so easily arrested, and none in which the tongue and inter-maxillary muscles can be as effectually supported, to say nothing of the probability of securing a new osseous deposit between the two laminæ of the periosteum of sufficient firmness to admit of a set of artificial teeth and render mastication easy and complete, beside bringing back the features almost to their natural state. The only incision made by M. Maisonneuve in all operations upon the lower jaw, whether of resection or ablation—is the perpendicular, as described—and has decided preferences over the angular or curved, first practiced by Dr. Deadrick, of this State, Tennessee, in 1810—two years after, by Dupuytren, and then by Cusack, Liston, Syme and Fergusson, of the British schools.

The advantages gained are, *first*, less liability of harming the parts torn by the action of the muscles, (masseter and buccinator)—*second*, less inflammation of the parts adjacent, and *third*, less deformity. With these preferable features—in operations requiring such skill and precaution—we would ask from the profession that impartial consideration which the importance of such a method demands.

I witnessed four partial excisions of the lower jaw in the same service; in each of which the periosteum was preserved, and so early as the fifth day, it had become firm and smooth; the cicatrix, down the margin of the genio, hyoideus muscle, was regular, and scarcely perceptible, while the jaws were round and full.

Three others, I had also the pleasure of witnessing, upon the superior maxilla ; two for scirrhous tumors situated upon the right side embracing the full space between the malar bone and a line corresponding with the second incisor, while the third occupied the left, involving two-thirds of the entire bone ; all of which was extracted, leaving exposed the tongue, a portion of the hyo-glossus muscle, the soft palate and all the teeth of the lower jaw—save the last molar.

These incisions were left open for the space of one month, and towards the sixth week, both patients quitted the hospital, having had adjusted, each, an artificial jaw of gutta-percha.

I witnessed, also, two total and one partial amputation of the Tongue for cancerous affections, by the same Surgeon, with his *ecraseur lineaire*, and in no case was the result unfavorable.

I mention these, to show the success which has followed each operation, notwithstanding his boldness and excessive thirst for *les maldies des Mâchoires.* In many respects, he is rough, and performs an operation as if foreman of a slaughter house, in consequence of which, he has received the sobriquet of *Chirurgien de l'Abattoir.*

